# Supplementary material for: Intracranial electrophysiological and structural basis of BOLD functional connectivity in human brain white matter
Source: Nat Commun. 2023 Jun 9;14:3414. doi: 10.1038/s41467-023-39067-3 (PMC10256794; doi:10.1038/s41467-023-39067-3)
Supplement: Supplementary file 1 — Supplementary Informaiton [file 41467_2023_39067_MOESM1_ESM.pdf]

Supplementary Information

Localization of electrode contacts

To localize the precise anatomical locations of the white matter contacts, we used the a priori White Matter Parcellation Map (WMPM) atlas[1,2], which includes 68 regions with 50 core and 18 peripheral white matter areas. First, we depicted a single participant (sub1, **Fig. S1a**) in the volume space. For better visualization, we projected all electrodes onto the same slice. We found a total of 47 white matter contacts for this participant, which were mainly localized in the temporal blade (28 contacts), inferior frontal blade (7 contacts), and parieto-temporal blade (5 contacts) (**Fig. S1a**). Next, we aggregated all 604 white matter contacts from all participants (**Fig. S1b**). Using the WMPM atlas, we found that the contacts were mainly localized in the temporal blade (288 contacts), frontal blade (122 contacts), parieto-temporal blade (54 contacts), anterior corona radiata (21 contacts), sagittal stratum (20 contacts), and posterior thalamic radiation (15 contacts).

Controlling for the distance between contacts

Prior studies have reported an association between distance and FC[3,4]; here, we evaluated the association between SEEG FC and Euclidean distance between contacts. We computed the Euclidean distance between each pair of contacts using the following formula: $\text{distance} = \sqrt{(x_1 - y_1)^2 + (x_2 - y_2)^2 + (x_3 - y_3)^2}$, where (x_1, x_2, x_3) and (y_1, y_2, y_3) are the coordinates of the centers of the two respective contacts. We constructed a distance matrix for each participant, with each element in the matrix quantifying the Euclidean distance between a pair of contacts. We found the SEEG FC was highly correlated with the distance across all participants in each frequency band. The median correlations between SEEG FC and distance ranged from r = -0.72 to r = -0.48 across all frequency bands (1-4 Hz: median r = -0.48; 4-8 Hz: median r = -0.58; 8-13 Hz: median r = -0.61; 13-30 Hz: median r = -0.59;

30-40 Hz: median r = -0.60; 40-70 Hz: median r = -0.72; 70-170 Hz: median r = -0.71; **Fig. S3**). See **Table S3** for r and p_{FDR} of the correlations for all participants at each frequency band. This result was similar to prior literature (See Fig. 4a in Betzel et al. 2019, Nat. Biomed. Eng.[3]). Similarly, BOLD FC also showed significant correlations with distance (median r = -0.3 across all participants), which was similar to the results reported in prior literature[4]. Therefore, we regressed out the distance from both matrices before examining the correlation between SEEG FC and BOLD FC throughout the manuscript. Similarly, as prior studies consistently reported an association between distance and structural connectivity[3,5], we also regressed out the distance from both matrices before evaluating the correlation between structural connectivity and other connectivity matrices. Notably, distance was regressed out in both the main analyses and all sensitivity analyses.

Sensitivity analysis for the association between SEEG and BOLD white matter FC

Analysis I: The impact of parameters for fMRI preprocessing and FC calculation

Here, we evaluated how fMRI preprocessing parameters impact the association between SEEG FC and BOLD FC. In our main analysis, we registered the coordinates of the SEEG contacts to the MNI space, which could lead to imprecise localization of the contacts. Here, we tested whether the results were consistent when examining the association between BOLD FC and SEEG FC in native space. We found that the median correlations between BOLD and SEEG white matter FC across all participants were higher than r = 0.17 in each frequency band (1-4 Hz: median r = 0.17; 4-8 Hz: median r = 0.22; 8-13 Hz: median r = 0.23; 13-30 Hz: median r = 0.30; 30-40 Hz: median r = 0.27; 40-70 Hz: median r = 0.26; 70-170 Hz: median r = 0.22; **Fig. S4a**). Of all the 112 correlations for all participants and all frequency bands, 92 were significant (p_{FDR} < 0.05) after FDR correction (**Table S4**). This result was similar to our main result.

Second, we used a bandpass filtering range of 0.01-0.2 Hz in the main results to include a wider frequency range of bold data in the white matter. Here, we evaluated a filtering range of 0.01-0.08 Hz, which is a commonly used range in the pre-processing of gray matter BOLD signals[6]. We found that the median correlations between BOLD and SEEG white matter FC across all participants were higher than $r = 0.16$ in each frequency band (1-4 Hz: median $r = 0.16$; 4-8 Hz: median $r = 0.20$; 8-13 Hz: median $r = 0.20$; 13-30 Hz: median $r = 0.26$; 30-40 Hz: median $r = 0.27$; 40-70 Hz: median $r = 0.24$; 70-170 Hz: median $r = 0.19$; **Fig. S4b**). Of all the 112 correlations for all participants and all frequency bands, 97 were significant ($p_{FDR} < 0.05$) after FDR correction (**Table S5**). This result was similar to our main result.

Third, we tested whether our results would be consistent after regressing out the global and CSF signals during fMRI preprocessing. We found that the median correlations between BOLD and SEEG white matter FC across all participants were higher than $r = 0.18$ in each frequency band (1-4 Hz: median $r = 0.18$; 4-8 Hz: median $r = 0.24$; 8-13 Hz: median $r = 0.24$; 13-30 Hz: median $r = 0.29$; 30-40 Hz: median $r = 0.31$; 40-70 Hz: median $r = 0.25$; 70-170 Hz: median $r = 0.22$; **Fig. S4c**). Of all the 112 correlations for all participants and all frequency bands, 111 were significant ($p_{FDR} < 0.05$) after FDR correction (**Table S6**). This result was similar to our main result.

Finally, we evaluated how the radius of the ROI for the BOLD FC calculation affected the correlation between SEEG FC and BOLD FC. In the main analysis, we defined the contact ROI comprising neighboring voxels adjacent to the surface and edge, which contained 19 voxels in total. Here, we tested the definition of ROI comprising neighboring voxels adjacent to the surface, which included 7 voxels, as well as the definition of ROI comprising neighbors adjacent to the surface, edge and vertex, which included 27 voxels. Using ROI definition of 7 voxels neighbors, we found that the median correlations between BOLD and SEEG white matter FC across all participants were higher than $r = 0.17$ in each

frequency band (1-4 Hz: median $r = 0.17$; 4-8 Hz: median $r = 0.21$; 8-13 Hz: median $r = 0.22$; 13-30 Hz: median $r = 0.27$; 30-40 Hz: median $r = 0.29$; 40-70 Hz: median $r = 0.27$; 70-170 Hz: median $r = 0.23$; **Fig. S5a**). Of all the 112 correlations for all participants and all frequency bands, 101 were significant ($p_{FDR} < 0.05$) after FDR correction (**Table S7**). This result was similar to our main result. We also obtained a consistent result with ROI defined as 27s voxel neighbors (See **Fig. S5b** and **Table S8**).

Analysis II: The impact of parameters for SEEG preprocessing and FC calculation

First, we evaluated whether the choice of window size for the SEEG data affected our main results. We used 10 consecutive segments of the SEEG time series data with a window size of 6 s, respectively, in our main results, and further tested a window size of 4 s and 8 s. Using a window size of 4 s, we found that the median correlations between BOLD and SEEG white matter FC across all participants were higher than $r = 0.16$ in each frequency band (1-4 Hz: median $r = 0.16$; 4-8 Hz: median $r = 0.26$; 8-13 Hz: median $r = 0.26$; 13-30 Hz: median $r = 0.30$; 30-40 Hz: median $r = 0.33$; 40-70 Hz: median $r = 28$; 70-170 Hz: median $r = 0.24$; **Fig. S6a**). Of all the 112 correlations for all participants and all frequency bands, 100 were significant ($p_{FDR} < 0.05$) after FDR correction (**Table S9**). This result was similar to our main result. We also found a consistent result with the 8 s window size (**Fig. S6b** and **Table S10**).

Second, in our main results, we evaluated the SEEG FC using the Pearson's correlation between the time series of two contacts. Here, we evaluated the coupling between SEEG and BOLD FC for a coherence-based SEEG FC. We found that the median correlations between BOLD and SEEG white matter FC across all participants was higher than $r = 0.22$ in each frequency band (1-4 Hz: median $r = 0.23$; 4-8 Hz: median $r = 0.22$; 8-13 Hz: median $r = 0.23$; 13-30 Hz: median $r = 0.24$; 30-40 Hz: median $r = 0.25$; 40-70 Hz: median $r = 0.27$; 70-170 Hz: median $r = 0.26$; see **Fig. S7**). Of all the 112 correlations for all participants and all frequency bands, 105 were significant ($p_{FDR} < 0.05$) after FDR correction

(**Table S11**). This result suggested that coherence-based SEEG FC also showed correlations with BOLD FC.

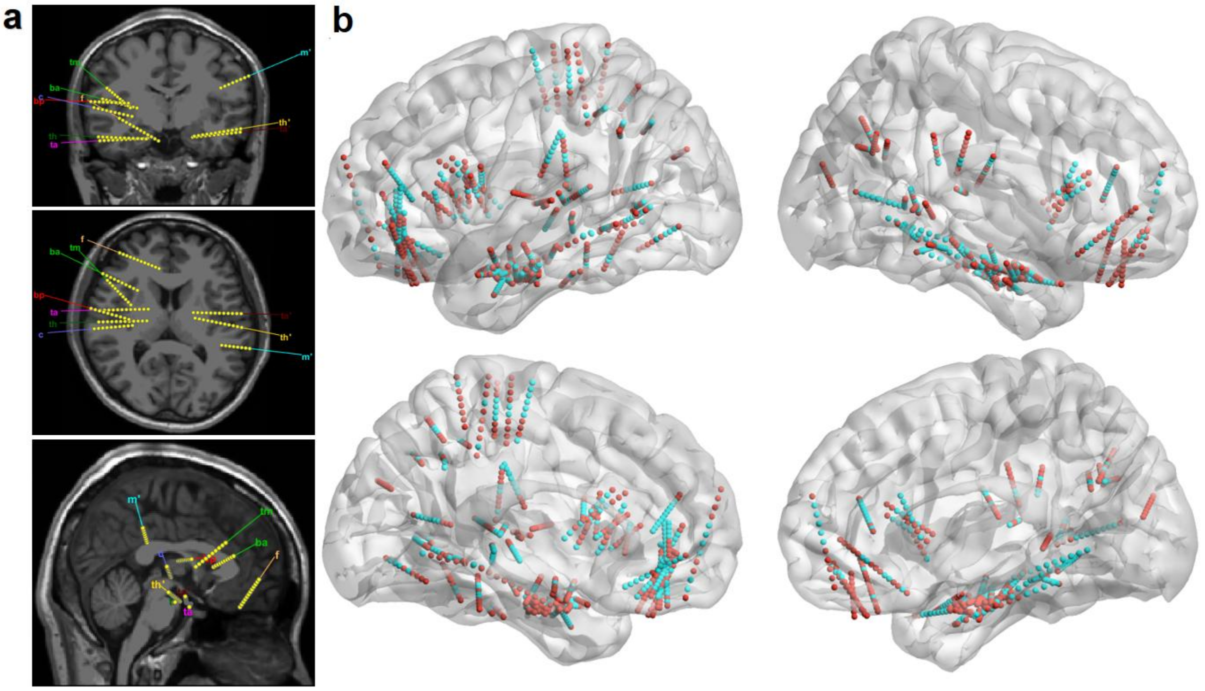

Fig. S1 Distribution of the contacts of SEEG implanted electrodes. a) The electrodes for one participant (sub1) in volume space. To facilitate visualization, we projected all electrodes to one slice of the brain. Visualization was performed with Brainstorm[7], which is documented and freely available for download online under the GNU general public license (http://neuroimage.usc.edu/brainstorm). **b**) The aggregation of all participants' electrodes visualized in surface space using BrainNet Viewer[8] (https://www.nitrc.org/projects/bnv). Red color indicates contacts in gray matter and green color indicates contacts in white matter. There are 604 white matter contacts in total. SEEG: stereotactic EEG.

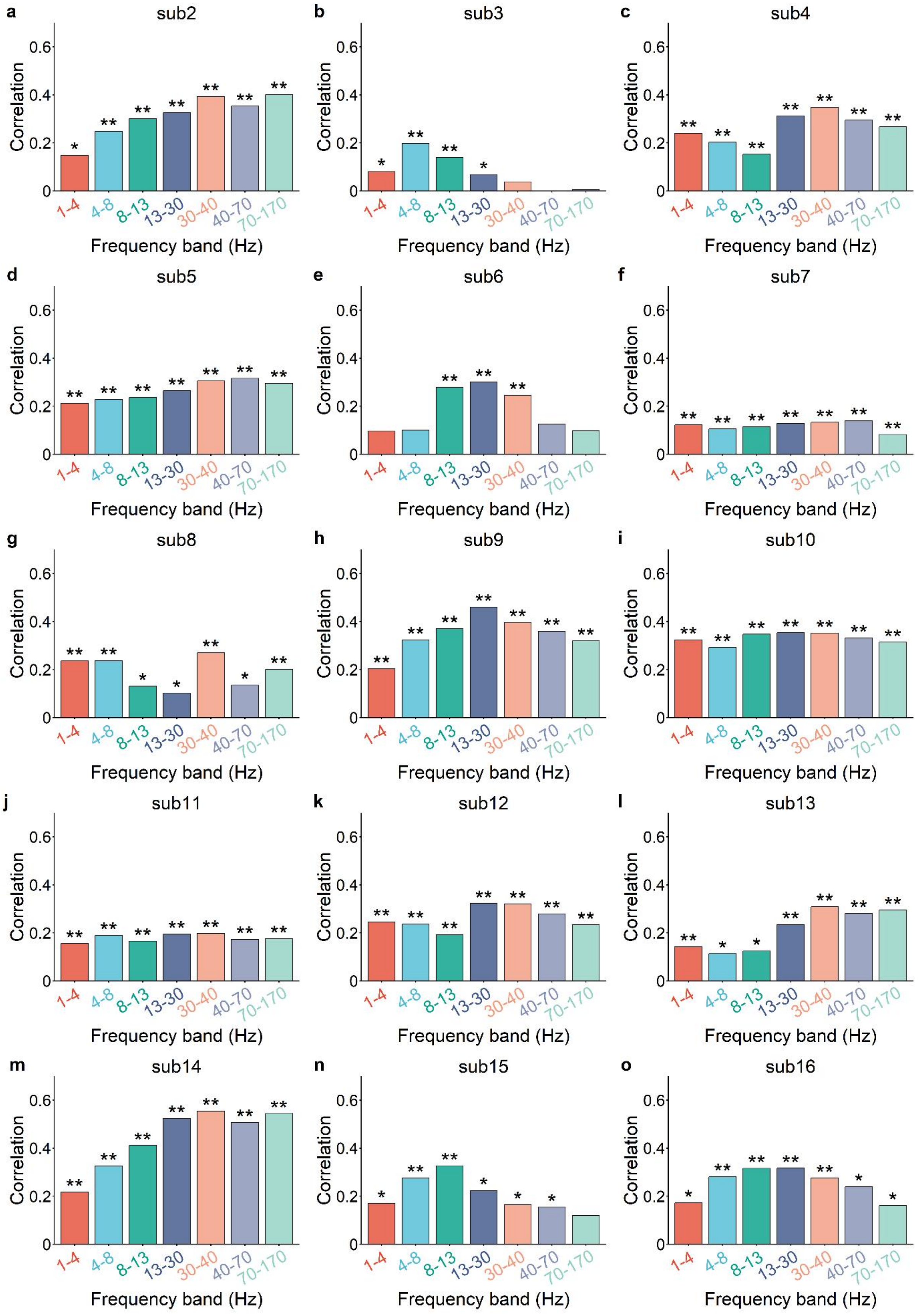

Fig. S2 Spearman's rank correlation between BOLD and SEEG FC in all frequency bands for 15 participants. The panels from **a)** to **o)** show the correlations between BOLD

and SEEG FC in sub2 to sub16, respectively, after regressing out the distances from both FC (See **Table S1** for participants' information). See **Fig. 1d** for the result of sub1. ** represents $p_{FDR} < 0.001$ and * represents $p_{FDR} < 0.05$ (See **Table S2** for detailed r and p_{FDR} value, two-sided). False discovery rate (FDR) correction was applied to account for multiple comparisons across all 16 participants and all frequency bands. See Table S1 for the number of each participant's functional connections, which defined the sample size of the correlation analysis for each participant. BOLD: blood-oxygenation-level-dependent; SEEG: stereotactic EEG; FC: functional connectivity.

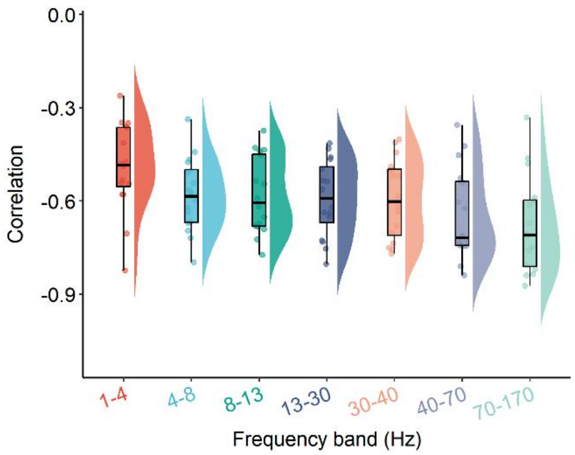

Fig. S3 Spearman's rank correlation between SEEG FC and contact distance. Each dot represents one participant. See **Table S3** for *r* and two-sided p_{FDR} of the correlations for all 16 participants at each frequency band. Boxes denote the 25th to 75th percentile and the median line. Whiskers extend 1.5 times the interquartile range from the edges of the box. False discovery rate (FDR) correction was applied to account for multiple comparisons across all 16 participants and all frequency bands. See Table S1 for the number of each participant's functional connections, which defined the sample size of the correlation analysis for each participant. SEEG: stereotactic EEG; FC: functional connectivity.

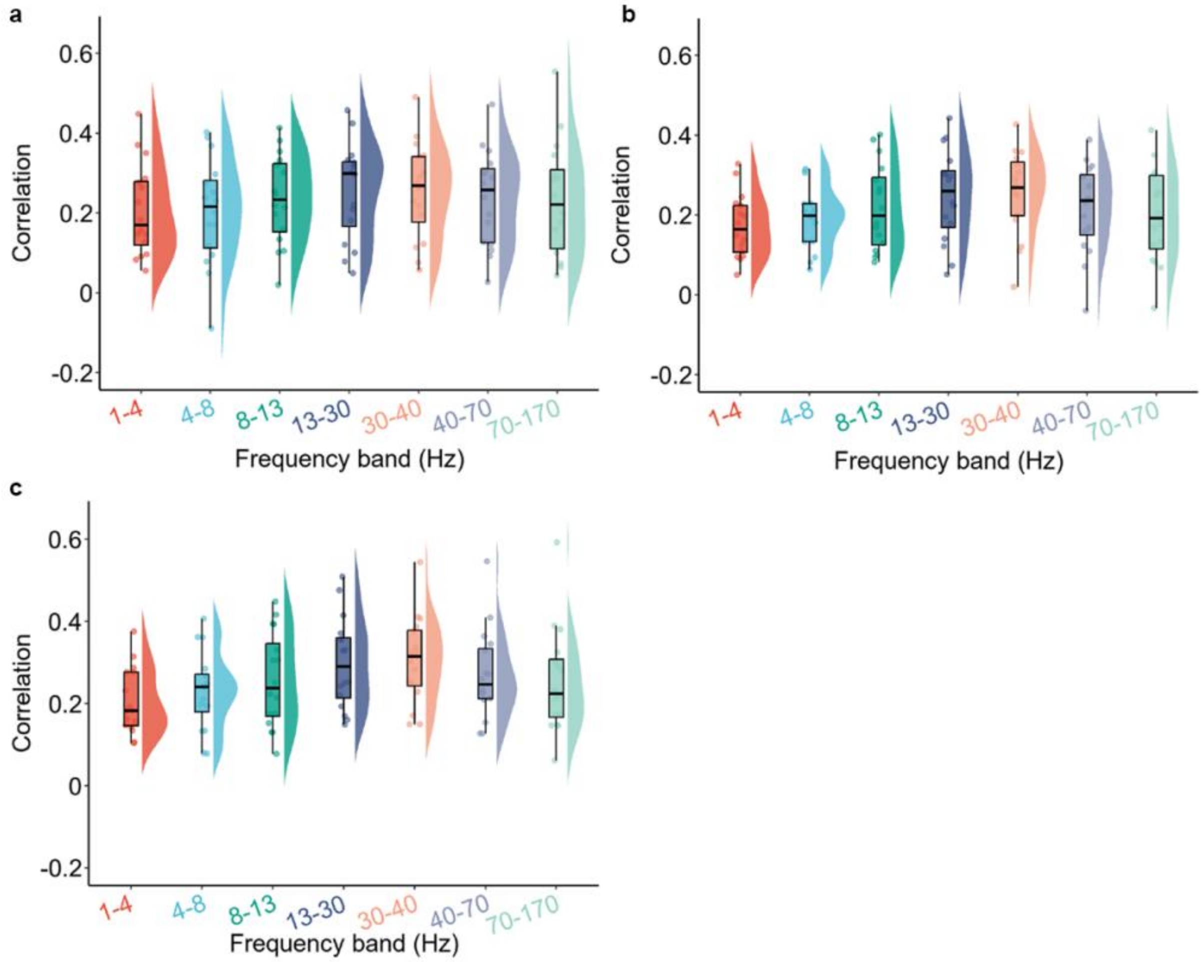

Fig. S4 The impact of fMRI preprocessing parameters in the Spearman's rank correlations between BOLD and SEEG FC. a) Evaluating the FC in native space, the Spearman's rank correlations between the BOLD and SEEG white matter FC were similar to the main results. Each dot represents one participant. See **Table S4** for *r* and two-sided p_{FDR} of the correlations for all 16 participants at each frequency band. **b)** With BOLD signals filtered using a frequency range of 0.01-0.08 Hz, the Spearman's rank correlations between the BOLD and SEEG white matter FC were similar to the main results. See **Table S5** for *r* and two-sided p_{FDR} of the correlations for all 16 participants at each frequency band. **c)** With global and CSF signals regressed out during fMRI preprocessing, the Spearman's rank correlations between BOLD and SEEG white matter FC were similar to the main results. See **Table S6** for *r* and two-sided p_{FDR} of the correlations for all 16 participants at each frequency band. Boxes denote the 25th to 75th percentile and the median line. Whiskers extend 1.5 times the interquartile range from the edges of the box. False discovery rate (FDR) correction was applied to account for multiple comparisons across all 16 participants and all frequency bands. See Table S1 for the number of each participant's functional connections, which defined the sample size of the correlation analysis for each participant. BOLD: blood-oxygenation-level-dependent; SEEG: stereotactic EEG; FC: functional connectivity.

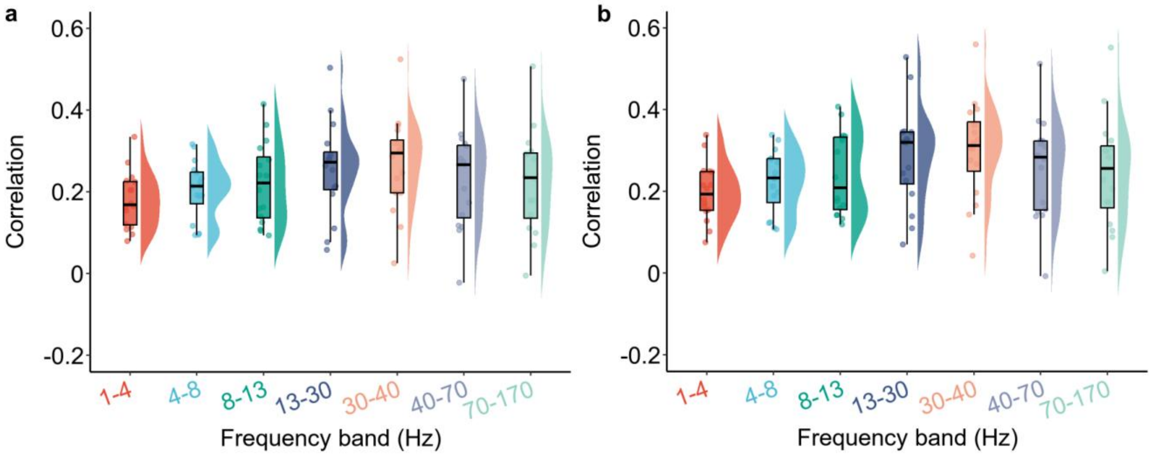

Fig. S5 Spearman's rank correlations between BOLD and SEEG FC were evaluated using different ROI size for BOLD FC calculation. a) With the definition of ROI comprising 7 neighboring voxels adjacent to the surface, the correlations between BOLD and SEEG white matter FC were similar to the main results. Each dot represents one participant. See **Table S7** for r and two-sided p_{FDR} of the correlations for all 16 participants at each frequency band. **b)** With the definition of ROI comprising 27 neighboring voxels adjacent to the surface, edge and vertex, the correlations between BOLD and SEEG white matter FC were similar to the main results. See **Table S8** for r and two-sided p_{FDR} of the correlations for all 16 participants at each frequency band. Boxes denote the 25th to 75th percentile and the median line. Whiskers extend 1.5 times the interquartile range from the edges of the box. False discovery rate (FDR) correction was applied to account for multiple comparisons across all 16 participants and all frequency bands. See Table S1 for the number of each participant's functional connections, which defined the sample size of the correlation analysis for each participant. BOLD: blood-oxygenation-level-dependent; SEEG: stereotactic EEG; FC: functional connectivity.

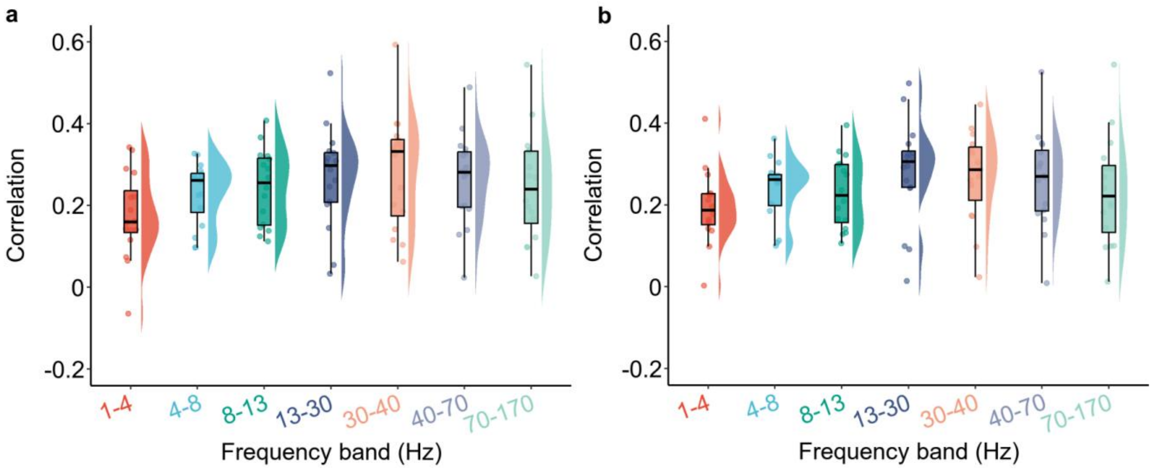

Fig. S6 Spearman's rank correlations between BOLD and SEEG FC were assessed using different window sizes for SEEG analysis. a) With a window size of 4 s, the correlations between BOLD and SEEG white matter FC were similar to the main results. Each dot represents one participant. See **Table S9** for r and two-sided p_{FDR} of the correlations for all 16 participants at each frequency band. **b)** With a window size of 8 s, the results were similar to the main results. Each dot represents one participant. See **Table S10** for r and two-sided p_{FDR} of the correlations for all 16 participants at each frequency band. Boxes denote the 25th to 75th percentile and the median line. Whiskers extend 1.5 times the interquartile range from the edges of the box. False discovery rate (FDR) correction was applied to account for multiple comparisons across all 16 participants and all frequency bands. See Table S1 for the number of each participant's functional connections, which defined the sample size of the correlation analysis for each participant. BOLD: blood-oxygenation-level-dependent; SEEG: stereotactic EEG; FC: functional connectivity.

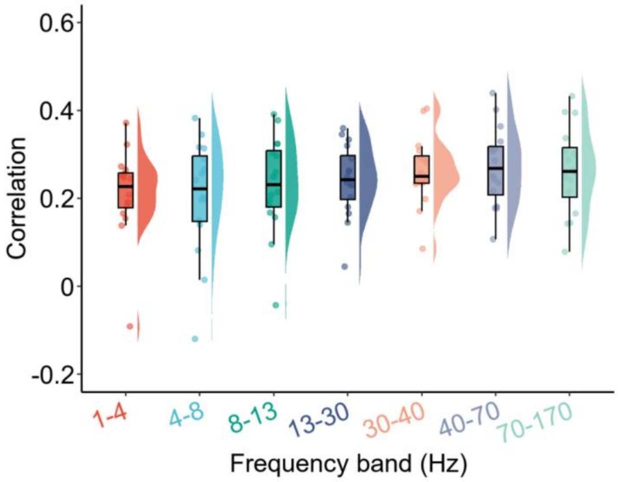

Fig. S7 Spearman's rank correlations between BOLD FC and coherence-based SEEG FC. Each dot represents one participant. See **Table S11** for *r* and two-sided p_{FDR} of the correlations for all 16 participants at each frequency band. Boxes denote the 25th to 75th percentile and the median line. Whiskers extend 1.5 times the interquartile range from the edges of the box. False discovery rate (FDR) correction was applied to account for multiple comparisons across all 16 participants and all frequency bands. See Table S1 for the number of each participant's functional connections, which defined the sample size of the correlation analysis for each participant. BOLD: blood-oxygenation-level-dependent; SEEG: stereotactic EEG; FC: functional connectivity.

Table S1 Subject demographics and characteristics.

ID	Epileptogenic Zone	Sex	Age	SR/Hz	ES	Contacts	WMC	FC	SC
1	Left temporal lobe, frontal base	F	29	2048	10	108	47	1081	511
2	Left orbitofrontal gyrus, left frontal pole	F	27	2048	7	76	30	435	103
3	Left posterior temporal cortex	M	31	2000	9	100	42	861	235
4	Left temporal lobe, hippocampus, amygdala	M	31	2048	8	92	36	630	161
5	Right medial temporal lobe	F	26	2000	7	80	31	465	219
6	Left temporal lobe	M	29	2000	9	72	23	253	105
7	Left temporal lobe, right frontal lobe	M	27	2000	12	146	71	2485	921
8	Left temporal lobe, hippocampal, amygdala	M	23	2000	9	77	34	561	88
9	Left hippocampus amygdala	M	33	2000	7	80	27	351	112
10	Right medial temporal lobe	F	37	2048	10	84	25	300	64
11	Paraventricular nodule	M	31	2000	10	96	63	1953	504
12	Left temporal lobe, hippocampus, amygdala	M	36	2000	12	150	49	1176	121
13	Left posterior temporal cortex	M	26	2000	10	94	38	703	88
14	Right temporal lobe	F	23	2048	8	88	24	276	80
15	Left frontal lobe	F	23	2048	6	58	21	210	68
16	Right hippocampus, amygdala, medial temporal lobe	F	19	2048	6	73	20	190	129

M, male; F, female; SR, sampling rate; ES, number of electrode shafts; Contacts, number of contacts; WMC, number of white matter contacts; FC, number of functional connections in white matter; SC, number of nonzero structural connectivity in white matter.

Table S2 The *r* and two-sided p_{FDR} values for the correlations between BOLD and SEEG FC for all seven different frequency bands and all 16 participants. The distance was regressed out from both FC matrices before evaluating the Spearman's rank correlation between them. Non-significant (threshold: p_{FDR} = 0.05) correlations were labeled with bold font. False discovery rate (FDR) correction was applied to account for multiple comparisons across all 16 participants and all frequency bands. BOLD: blood-oxygenation-level-dependent; SEEG: stereotactic EEG; FC: functional connectivity.

ID	r/p_{FDR}	**1-4Hz**	**4-8Hz**	**8-13Hz**	**13-30Hz**	**30-40Hz**	**40-70Hz**	**70-170Hz**
sub01	*r*	0.32	0.13	0.17	0.32	0.37	0.32	0.33
	p	1.2e-25	4.1e-05	1.7e-08	2.7e-25	6.5e-35	2.7e-25	1.2e-27
sub02	*r*	0.15	0.25	0.30	0.32	0.39	0.35	0.40
	p	2.3e-03	2.7e-07	3.7e-10	1.1e-11	1.1e-16	1.4e-13	2.4e-17
sub03	*r*	0.08	0.20	0.14	0.07	**0.04**	**-0.01**	**0.00**
	p	2.1e-02	9.7e-09	5.9e-05	4.9e-02	**2.8e-01**	**7.7e-01**	**8.9e-01**
sub04	*r*	0.24	0.20	0.15	0.31	0.35	0.29	0.27
	p	2.0e-09	4.3e-07	1.7e-04	4.5e-15	1.8e-18	1.9e-13	2.9e-11
sub05	*r*	0.21	0.23	0.24	0.26	0.31	0.32	0.30
	p	5.8e-06	1.0e-06	4.2e-07	1.5e-08	4.8e-11	1.0e-11	1.9e-10
sub06	*r*	**0.10**	**0.10**	0.28	0.30	0.24	**0.12**	**0.10**
	p	**1.3e-01**	**1.2e-01**	9.9e-06	1.9e-06	1.1e-04	**5.1e-02**	**1.3e-01**
sub07	*r*	0.12	0.10	0.11	0.13	0.13	0.14	0.08
	p	2.1e-09	2.7e-07	2.1e-08	3.1e-10	9.1e-11	8.3e-12	5.9e-05
sub08	*r*	0.24	0.24	0.13	0.10	0.27	0.14	0.20
	p	2.9e-08	2.4e-08	2.4e-03	1.9e-02	2.1e-10	1.6e-03	2.6e-06
sub09	*r*	0.20	0.32	0.37	0.46	0.40	0.36	0.32
	p	1.7e-04	1.3e-09	2.7e-12	8.4e-19	4.9e-14	1.1e-11	2.0e-09
sub10	*r*	0.32	0.29	0.35	0.35	0.35	0.33	0.31
	p	1.8e-08	4.2e-07	1.5e-09	7.2e-10	8.3e-10	8.2e-09	5.0e-08
sub11	*r*	0.16	0.19	0.17	0.19	0.20	0.17	0.18
	p	1.4e-11	1.8e-16	7.3e-13	2.4e-17	7.0e-18	4.9e-14	2.3e-14
sub12	*r*	0.25	0.24	0.19	0.32	0.32	0.28	0.23
	p	7.3e-17	8.8e-16	7.6e-11	5.7e-28	7.6e-28	1.7e-21	2.9e-15
sub13	*r*	0.14	0.11	0.12	0.23	0.31	0.28	0.29
	p	2.0e-04	3.2e-03	1.2e-03	7.2e-10	3.0e-16	1.2e-13	7.2e-15
sub14	*r*	0.22	0.33	0.41	0.52	0.55	0.51	0.54
	p	3.4e-04	5.0e-08	3.8e-12	8.2e-20	1.7e-22	1.6e-18	1.4e-21
sub15	*r*	0.17	0.28	0.33	0.22	0.16	0.15	**0.12**
	p	1.6e-02	6.9e-05	2.0e-06	1.3e-03	1.9e-02	2.8e-02	**9.0e-02**
sub16	*r*	0.17	0.28	0.32	0.32	0.28	0.24	0.16
	p	2.0e-02	1.2e-04	1.3e-05	1.3e-05	1.6e-04	1.1e-03	2.9e-02

Table S3 The *r* and two-sided p_{FDR} values for the Spearman's rank correlations between SEEG FC and contact distance. False discovery rate (FDR) correction was applied to account for multiple comparisons across all 16 participants and all frequency bands. SEEG: stereotactic EEG; FC: functional connectivity.

ID	r/p_{FDR}	**1-4Hz**	**4-8Hz**	**8-13Hz**	**13-30Hz**	**30-40Hz**	**40-70Hz**	**70-170Hz**
sub01	*r*	-0.58	-0.70	-0.69	-0.75	-0.62	-0.74	-0.76
	p	1.6e-97	3.0e-156	8.4e-154	1.4e-197	1.1e-114	2.4e-189	1.8e-203
sub02	*r*	-0.55	-0.56	-0.62	-0.73	-0.75	-0.75	-0.81
	p	6.3e-35	8.9e-38	6.7e-47	2.8e-72	1.3e-79	6.5e-78	7.9e-101
sub03	*r*	-0.48	-0.63	-0.44	-0.41	-0.44	-0.42	-0.46
	p	2.1e-50	1.1e-97	1.3e-41	8.5e-37	1.4e-42	1.7e-38	9.1e-47
sub04	*r*	-0.41	-0.47	-0.55	-0.53	-0.52	-0.54	-0.61
	p	2.5e-27	3.4e-35	7.5e-50	1.5e-47	7.5e-45	6.4e-49	1.2e-65
sub05	*r*	-0.36	-0.44	-0.37	-0.50	-0.74	-0.62	-0.67
	p	6.2e-16	1.3e-23	8.2e-17	2.1e-30	4.2e-80	3.1e-51	2.0e-61
sub06	*r*	-0.48	-0.67	-0.59	-0.56	-0.52	-0.71	-0.59
	p	1.0e-15	8.1e-34	3.0e-25	1.5e-22	1.2e-18	2.2e-40	4.8e-25
sub07	*r*	-0.35	-0.34	-0.44	-0.43	-0.41	-0.45	-0.48
	p	3.6e-71	1.3e-66	5.2e-115	1.4e-112	6.1e-102	1.9e-125	1.3e-142
sub08	*r*	-0.36	-0.54	-0.68	-0.64	-0.49	-0.53	-0.60
	p	1.1e-18	5.5e-44	5.7e-78	6.8e-65	4.8e-35	2.7e-41	8.6e-56
sub09	*r*	-0.54	-0.60	-0.65	-0.62	-0.68	-0.75	-0.75
	p	4.2e-28	3.9e-35	1.1e-43	2.2e-38	8.2e-49	6.2e-63	2.4e-64
sub10	*r*	-0.35	-0.51	-0.45	-0.46	-0.70	-0.72	-0.71
	p	4.5e-10	5.7e-21	1.6e-16	1.9e-17	1.2e-45	8.6e-50	2.2e-46
sub11	*r*	-0.82	-0.80	-0.77	-0.80	-0.76	-0.81	-0.84
	p	0.0e+00	0.0e+00	0.0e+00	0.0e+00	0.0e+00	0.0e+00	0.0e+00
sub12	*r*	-0.26	-0.62	-0.54	-0.64	-0.59	-0.74	-0.82
	p	7.8e-20	7.2e-126	5.8e-88	2.1e-135	1.8e-108	6.3e-202	4.5e-286
sub13	*r*	-0.58	-0.57	-0.68	-0.65	-0.64	-0.73	-0.87
	p	9.6e-64	5.2e-62	6.2e-96	2.2e-85	3.8e-80	4.9e-116	1.4e-219
sub14	*r*	-0.49	-0.48	-0.43	-0.46	-0.50	-0.60	-0.71
	p	6.8e-18	6.0e-17	9.1e-14	8.0e-16	7.6e-19	8.9e-29	5.8e-44
sub15	*r*	-0.53	-0.72	-0.67	-0.54	-0.40	-0.36	-0.33
	p	8.6e-17	1.5e-34	6.9e-29	4.7e-17	1.6e-09	1.2e-07	8.9e-07
sub16	*r*	-0.71	-0.67	-0.72	-0.73	-0.77	-0.84	-0.84
	p	8.7e-30	4.0e-26	5.6e-32	5.2e-33	3.2e-38	3.2e-51	1.7e-50

Table S4 The *r* and two-sided p_{FDR} values for the correlations between BOLD and SEEG FC evaluated in native space. The distance was regressed out from both FC matrices before evaluating the Spearman's rank correlation between them. Non-significant (threshold: p_{FDR} = 0.05) correlations were labeled with bold font. False discovery rate (FDR) correction was applied to account for multiple comparisons across all 16 participants and all frequency bands. BOLD: blood-oxygenation-level-dependent; SEEG: stereotactic EEG; FC: functional connectivity.

ID	r/p_{FDR}	**1-4Hz**	**4-8Hz**	**8-13Hz**	**13-30Hz**	**30-40Hz**	**40-70Hz**	**70-170Hz**
sub01	*r*	0.37	**0.05**	0.16	0.30	0.37	0.29	0.30
	p	7.2e-35	**1.2e-01**	2.7e-07	3.1e-22	4.5e-35	3.1e-21	4.1e-22
sub02	*r*	0.15	0.30	0.38	0.34	0.39	0.37	0.42
	p	2.5e-03	5.3e-10	6.1e-16	4.7e-13	1.1e-16	5.4e-15	6.5e-19
sub03	*r*	0.09	0.17	0.13	0.08	**0.06**	**0.03**	**0.04**
	p	8.9e-03	7.8e-07	1.1e-04	2.6e-02	**1.1e-01**	**4.3e-01**	**2.1e-01**
sub04	*r*	0.23	0.23	0.22	0.33	0.29	0.28	0.24
	p	1.4e-08	8.8e-09	5.4e-08	4.3e-17	3.2e-13	4.9e-12	1.2e-09
sub05	*r*	0.18	0.25	0.30	0.32	0.30	0.32	0.28
	p	9.7e-05	7.0e-08	6.7e-11	5.8e-12	7.2e-11	2.1e-12	9.8e-10
sub06	*r*	**0.13**	**0.09**	0.21	0.21	0.20	**0.12**	**0.06**
	p	**5.2e-02**	**1.4e-01**	8.5e-04	1.1e-03	2.4e-03	**6.2e-02**	**3.2e-01**
sub07	*r*	0.08	0.08	0.10	0.10	0.12	0.13	0.10
	p	5.9e-05	1.6e-04	9.2e-07	1.1e-06	3.0e-09	7.4e-10	1.2e-06
sub08	*r*	0.45	0.40	0.35	0.33	0.34	0.30	0.29
	p	1.7e-27	4.0e-22	3.0e-17	6.7e-15	4.5e-16	1.6e-12	9.8e-12
sub09	*r*	0.35	0.39	0.41	0.42	0.34	0.36	0.37
	p	3.2e-11	1.1e-13	2.1e-15	3.8e-16	7.3e-11	1.4e-11	2.8e-12
sub10	*r*	**0.06**	**-0.09**	**0.02**	**0.05**	**0.08**	**0.09**	**0.07**
	p	**3.4e-01**	**1.4e-01**	**7.3e-01**	**4.0e-01**	**2.0e-01**	**1.2e-01**	**2.2e-01**
sub11	*r*	0.16	0.17	0.20	0.22	0.22	0.20	0.18
	p	7.8e-12	7.2e-14	9.6e-18	8.8e-22	1.4e-21	1.2e-17	1.2e-14
sub12	*r*	0.28	0.24	0.25	0.30	0.28	0.24	0.20
	p	4.5e-21	4.7e-16	3.9e-17	8.9e-25	5.1e-21	3.8e-16	1.1e-11
sub13	*r*	0.26	0.12	0.10	0.18	0.23	0.31	0.34
	p	3.4e-12	2.4e-03	6.9e-03	2.0e-06	1.8e-09	3.8e-16	3.6e-20
sub14	*r*	0.15	0.28	0.33	0.46	0.49	0.47	0.55
	p	1.9e-02	4.9e-06	2.9e-08	3.9e-15	2.4e-17	4.2e-16	2.8e-22
sub15	*r*	**0.10**	0.20	0.26	**0.12**	**0.11**	**0.11**	**0.11**
	p	**1.8e-01**	4.4e-03	2.5e-04	**9.5e-02**	**1.1e-01**	**1.4e-01**	**1.2e-01**
sub16	*r*	0.29	0.37	0.32	0.32	0.26	0.18	0.16
	p	9.1e-05	2.8e-07	9.4e-06	1.1e-05	3.5e-04	1.8e-02	3.4e-02

Table S5 The r and two-sided p_{FDR} values for the correlations between BOLD and SEEG FC evaluated using a bandpass filtering rang of 0.01-0.08 Hz in fMRI data processing. The distance was regressed out from both FC matrices before evaluating the Spearman's rank correlation between them. Non-significant (threshold: $p_{FDR} = 0.05$) correlations were labeled with bold font. False discovery rate (FDR) correction was applied to account for multiple comparisons across all 16 participants and all frequency bands. BOLD: blood-oxygenation-level-dependent; SEEG: stereotactic EEG; FC: functional connectivity.

ID	r/p_{FDR}	1-4Hz	4-8Hz	8-13Hz	13-30Hz	30-40Hz	40-70Hz	70-170Hz
sub01	*r*	0.30	0.09	0.15	0.29	0.33	0.30	0.31
	p	2.9e-23	2.6e-03	1.3e-06	6.7e-21	8.6e-27	4.3e-22	7.3e-24
sub02	*r*	0.13	0.23	0.27	0.30	0.34	0.32	0.35
	p	1.0e-02	2.2e-06	3.5e-08	4.3e-10	1.2e-12	6.8e-11	4.0e-13
sub03	*r*	**0.05**	0.20	0.13	0.07	**0.02**	**-0.04**	**-0.03**
	p	**1.5e-01**	1.5e-08	1.9e-04	3.8e-02	**5.5e-01**	**2.5e-01**	**3.4e-01**
sub04	*r*	0.18	0.13	0.10	0.23	0.25	0.23	0.21
	p	8.5e-06	1.0e-03	1.4e-02	1.0e-08	6.0e-10	1.0e-08	3.0e-07
sub05	*r*	0.20	0.21	0.22	0.22	0.27	0.27	0.26
	p	3.1e-05	8.8e-06	3.5e-06	2.1e-06	1.4e-08	5.8e-09	3.4e-08
sub06	*r*	**0.09**	0.13	0.32	0.34	0.29	0.16	**0.09**
	p	**1.7e-01**	3.9e-02	4.8e-07	8.4e-08	4.6e-06	1.4e-02	**1.8e-01**
sub07	*r*	0.10	0.08	0.11	0.12	0.12	0.12	0.07
	p	1.9e-06	6.7e-05	6.0e-08	3.2e-09	4.1e-09	2.1e-09	8.2e-04
sub08	*r*	0.24	0.21	0.10	**0.05**	0.20	**0.07**	0.13
	p	1.0e-08	7.8e-07	2.9e-02	**2.4e-01**	2.6e-06	**1.0e-01**	2.2e-03
sub09	*r*	0.22	0.32	0.40	0.44	0.36	0.34	0.30
	p	3.8e-05	3.7e-09	4.3e-14	3.4e-17	2.2e-11	2.8e-10	2.2e-08
sub10	*r*	0.33	0.31	0.39	0.39	0.36	0.32	0.30
	p	1.3e-08	6.6e-08	1.4e-11	1.5e-11	4.2e-10	2.2e-08	2.4e-07
sub11	*r*	0.15	0.20	0.17	0.20	0.19	0.17	0.17
	p	1.4e-10	4.1e-18	4.3e-13	3.7e-17	3.9e-16	5.4e-13	3.5e-13
sub12	*r*	0.20	0.18	0.18	0.30	0.31	0.24	0.18
	p	1.1e-11	1.2e-09	1.7e-09	9.5e-25	3.7e-25	4.9e-16	2.9e-09
sub13	*r*	0.09	**0.06**	0.08	0.18	0.27	0.24	0.25
	p	1.4e-02	**9.9e-02**	3.5e-02	3.0e-06	1.0e-12	3.5e-10	6.7e-11
sub14	*r*	0.23	0.31	0.36	0.39	0.43	0.39	0.41
	p	1.8e-04	4.3e-07	2.0e-09	5.2e-11	7.1e-13	8.2e-11	4.9e-12
sub15	*r*	**0.11**	0.20	0.26	0.14	**0.11**	**0.11**	**0.08**
	p	**1.2e-01**	5.4e-03	2.3e-04	4.7e-02	**1.2e-01**	**1.2e-01**	**2.5e-01**
sub16	*r*	**0.14**	0.23	0.29	0.30	0.25	0.20	**0.12**
	p	**6.4e-02**	2.1e-03	8.5e-05	4.7e-05	5.8e-04	8.2e-03	**9.7e-02**

Table S6 The *r* and two-sided p_{FDR} values for the correlations between BOLD and SEEG FC evaluated with global and CSF signals regressed out in fMRI preprocessing. The distance was regressed out from both FC matrices before evaluating the Spearman's rank correlation between them. Non-significant (threshold: $p_{FDR} = 0.05$) correlations were labeled with bold font. False discovery rate (FDR) correction was applied to account for multiple comparisons across all 16 participants and all frequency bands. BOLD: blood-oxygenation-level-dependent; SEEG: stereotactic EEG; FC: functional connectivity.

ID	r/p_{FDR}	**1-4Hz**	**4-8Hz**	**8-13Hz**	**13-30Hz**	**30-40Hz**	**40-70Hz**	**70-170Hz**
sub01	*r*	0.29	0.08	0.15	0.25	0.25	0.23	0.23
	p	6.0e-21	1.1e-02	7.5e-07	2.0e-16	8.0e-16	2.1e-13	4.7e-14
sub02	*r*	0.18	0.27	0.31	0.33	0.38	0.34	0.38
	p	1.9e-04	2.6e-08	1.5e-10	4.0e-12	2.0e-15	3.8e-13	9.9e-16
sub03	*r*	0.11	0.20	0.21	0.16	0.15	0.13	0.15
	p	2.2e-03	7.9e-09	3.8e-10	3.5e-06	1.6e-05	2.2e-04	1.7e-05
sub04	*r*	0.28	0.24	0.18	0.35	0.39	0.33	0.30
	p	4.0e-12	2.5e-09	1.2e-05	2.7e-19	1.5e-22	1.3e-16	3.2e-14
sub05	*r*	0.14	0.13	0.13	0.17	0.25	0.21	0.17
	p	2.3e-03	4.6e-03	5.5e-03	3.1e-04	4.3e-08	5.5e-06	2.2e-04
sub06	*r*	0.19	0.25	0.39	0.37	0.33	0.23	0.17
	p	2.5e-03	8.5e-05	1.6e-10	2.1e-09	1.2e-07	3.4e-04	6.3e-03
sub07	*r*	0.10	0.08	0.08	0.15	0.15	0.13	0.06
	p	4.4e-07	1.1e-04	1.5e-04	2.7e-13	1.8e-13	3.7e-10	2.3e-03
sub08	*r*	0.16	0.20	0.25	0.24	0.34	0.26	0.29
	p	2.5e-04	4.8e-06	3.0e-09	1.0e-08	8.9e-16	7.4e-10	4.4e-12
sub09	*r*	0.23	0.36	0.42	0.48	0.41	0.36	0.32
	p	1.7e-05	6.1e-12	1.8e-15	2.8e-20	8.5e-15	4.7e-12	9.1e-10
sub10	*r*	0.38	0.36	0.39	0.41	0.41	0.41	0.39
	p	3.7e-11	1.9e-10	4.0e-12	2.1e-13	3.3e-13	4.1e-13	5.8e-12
sub11	*r*	0.18	0.21	0.17	0.22	0.23	0.21	0.22
	p	1.3e-15	3.3e-20	3.2e-14	1.2e-21	3.7e-23	8.2e-20	2.6e-21
sub12	*r*	0.28	0.24	0.22	0.36	0.33	0.27	0.21
	p	4.5e-21	1.4e-16	3.2e-14	1.9e-34	6.7e-29	9.1e-21	4.9e-13
sub13	*r*	0.15	0.13	0.13	0.25	0.30	0.27	0.28
	p	1.2e-04	4.5e-04	6.3e-04	3.7e-11	1.2e-15	6.2e-13	1.4e-13
sub14	*r*	0.31	0.41	0.45	0.51	0.54	0.55	0.59
	p	1.6e-07	4.8e-12	1.7e-14	8.9e-19	1.4e-21	1.2e-21	5.7e-26
sub15	*r*	**0.13**	0.24	0.31	0.19	0.17	0.15	0.15
	p	**5.2e-02**	4.6e-04	9.6e-06	5.4e-03	1.4e-02	2.6e-02	3.5e-02
sub16	*r*	0.16	0.29	0.33	0.33	0.28	0.23	0.15
	p	2.6e-02	9.1e-05	4.8e-06	5.5e-06	1.0e-04	1.4e-03	4.3e-02

Table S7 The *r* and two-sided p_{FDR} values for the correlations between BOLD and SEEG FC evaluated using a ROI with neighboring 7 voxels adjacent to surface in BOLD FC analysis. The distance was regressed out from both FC matrices before evaluating the Spearman's rank correlation between them. Non-significant (threshold: p_{FDR} = 0.05) correlations were labeled with bold font. False discovery rate (FDR) correction was applied to account for multiple comparisons across all 16 participants and all frequency bands. BOLD: blood-oxygenation-level-dependent; SEEG: stereotactic EEG; FC: functional connectivity.

ID	r/p_{FDR}	**1-4Hz**	**4-8Hz**	**8-13Hz**	**13-30Hz**	**30-40Hz**	**40-70Hz**	**70-170Hz**
sub01	*r*	0.27	0.12	0.16	0.28	0.32	0.28	0.29
	p	1.2e-18	1.8e-04	2.8e-07	1.0e-19	1.2e-24	9.2e-20	1.9e-21
sub02	*r*	0.12	0.21	0.26	0.29	0.35	0.31	0.36
	p	1.4e-02	1.1e-05	4.0e-08	1.7e-09	2.2e-13	8.5e-11	3.4e-14
sub03	*r*	0.08	0.19	0.13	**0.06**	**0.03**	**-0.02**	**-0.01**
	p	2.3e-02	2.7e-08	3.1e-04	**9.4e-02**	**4.6e-01**	**5.2e-01**	**8.8e-01**
sub04	*r*	0.22	0.19	0.14	0.28	0.32	0.27	0.24
	p	2.6e-08	2.8e-06	5.8e-04	1.2e-12	1.7e-15	3.5e-11	4.4e-09
sub05	*r*	0.20	0.22	0.24	0.28	0.30	0.32	0.29
	p	1.3e-05	1.7e-06	2.6e-07	2.0e-09	8.5e-11	6.7e-12	3.9e-10
sub06	*r*	**0.10**	**0.10**	0.24	0.25	0.20	**0.11**	**0.10**
	p	**1.3e-01**	**1.3e-01**	1.8e-04	6.9e-05	2.1e-03	**9.7e-02**	**1.2e-01**
sub07	*r*	0.11	0.10	0.09	0.11	0.11	0.12	0.07
	p	2.2e-07	4.6e-06	7.7e-06	1.0e-07	3.8e-08	1.5e-08	8.5e-04
sub08	*r*	0.20	0.22	0.11	**0.08**	0.25	0.11	0.18
	p	1.7e-06	4.4e-07	1.2e-02	**7.5e-02**	7.2e-09	8.5e-03	3.2e-05
sub09	*r*	0.17	0.28	0.32	0.40	0.37	0.33	0.30
	p	1.6e-03	2.4e-07	1.2e-09	3.8e-14	4.4e-12	6.1e-10	2.3e-08
sub10	*r*	0.33	0.31	0.36	0.37	0.36	0.34	0.32
	p	6.9e-09	8.1e-08	2.2e-10	1.9e-10	2.9e-10	3.3e-09	3.1e-08
sub11	*r*	0.16	0.19	0.16	0.20	0.20	0.17	0.18
	p	9.2e-13	1.3e-16	2.2e-12	2.7e-17	9.3e-18	5.8e-14	7.3e-15
sub12	*r*	0.24	0.23	0.20	0.32	0.30	0.27	0.23
	p	1.7e-15	3.4e-15	4.6e-12	5.0e-27	1.4e-24	3.1e-20	4.5e-15
sub13	*r*	0.11	0.09	0.10	0.21	0.29	0.27	0.28
	p	3.0e-03	1.5e-02	6.6e-03	2.2e-08	3.9e-14	2.7e-12	1.9e-13
sub14	*r*	0.23	0.32	0.41	0.50	0.52	0.48	0.51
	p	2.1e-04	1.5e-07	2.6e-12	3.8e-18	1.0e-19	4.0e-16	2.5e-18
sub15	*r*	0.15	0.25	0.31	0.21	0.15	0.14	**0.11**
	p	2.9e-02	2.9e-04	9.5e-06	2.9e-03	2.9e-02	4.3e-02	**1.1e-01**
sub16	*r*	0.16	0.25	0.28	0.26	0.23	0.21	**0.14**
	p	3.0e-02	8.0e-04	1.5e-04	3.1e-04	1.6e-03	4.9e-03	**5.3e-02**

Table S8 The *r* and two-sided p_{FDR} values for the correlations between BOLD and SEEG FC evaluated using a ROI with neighboring 27 voxels adjacent to surface, edge, and vertex in BOLD FC analysis. The distance was regressed out from both FC matrices before evaluating the Spearman's rank correlation between them. Non-significant (threshold: p_{FDR} = 0.05) correlations were labeled with bold font. False discovery rate (FDR) correction was applied to account for multiple comparisons across all 16 participants and all frequency bands. BOLD: blood-oxygenation-level-dependent; SEEG: stereotactic EEG; FC: functional connectivity.

ID	r/p_{FDR}	**1-4Hz**	**4-8Hz**	**8-13Hz**	**13-30Hz**	**30-40Hz**	**40-70Hz**	**70-170Hz**
sub01	*r*	0.34	0.12	0.18	0.33	0.39	0.32	0.34
	p	8.1e-29	5.6e-05	1.1e-08	9.4e-27	3.0e-39	1.0e-26	4.2e-29
sub02	*r*	0.16	0.27	0.32	0.35	0.41	0.37	0.42
	p	7.2e-04	1.8e-08	1.0e-11	4.1e-13	1.6e-18	5.2e-15	3.5e-19
sub03	*r*	0.08	0.20	0.14	0.07	**0.04**	**-0.01**	**0.00**
	p	2.9e-02	8.6e-09	3.9e-05	4.4e-02	**2.2e-01**	**8.3e-01**	**8.9e-01**
sub04	*r*	0.25	0.21	0.16	0.33	0.36	0.31	0.28
	p	4.3e-10	1.3e-07	7.5e-05	1.4e-16	5.7e-20	8.2e-15	1.3e-12
sub05	*r*	0.22	0.23	0.23	0.26	0.31	0.32	0.30
	p	4.1e-06	7.3e-07	5.0e-07	2.9e-08	3.8e-11	8.5e-12	1.1e-10
sub06	*r*	**0.10**	**0.11**	0.30	0.32	0.27	0.14	**0.10**
	p	**1.1e-01**	**9.5e-02**	2.3e-06	3.0e-07	2.3e-05	3.0e-02	**1.0e-01**
sub07	*r*	0.13	0.11	0.12	0.14	0.14	0.15	0.09
	p	3.3e-10	5.7e-08	4.6e-09	8.7e-12	2.2e-12	1.7e-13	1.7e-05
sub08	*r*	0.25	0.25	0.14	0.11	0.28	0.14	0.21
	p	4.5e-09	7.6e-09	9.6e-04	1.1e-02	7.9e-11	9.6e-04	1.3e-06
sub09	*r*	0.21	0.34	0.39	0.48	0.40	0.37	0.32
	p	6.5e-05	1.8e-10	1.7e-13	1.5e-20	2.4e-14	4.5e-12	1.1e-09
sub10	*r*	0.31	0.28	0.34	0.34	0.34	0.32	0.31
	p	5.5e-08	1.2e-06	4.5e-09	1.8e-09	2.0e-09	1.8e-08	1.0e-07
sub11	*r*	0.15	0.19	0.17	0.19	0.20	0.17	0.17
	p	4.2e-11	2.0e-16	4.0e-13	3.1e-17	1.4e-17	5.7e-14	5.2e-14
sub12	*r*	0.25	0.23	0.18	0.32	0.33	0.28	0.23
	p	3.9e-17	1.7e-15	5.4e-10	6.1e-28	4.2e-29	1.9e-21	8.2e-15
sub13	*r*	0.15	0.12	0.13	0.24	0.32	0.29	0.30
	p	5.3e-05	1.3e-03	5.3e-04	1.4e-10	3.4e-17	2.4e-14	2.7e-15
sub14	*r*	0.21	0.33	0.41	0.53	0.56	0.51	0.55
	p	6.8e-04	5.4e-08	5.5e-12	3.1e-20	5.2e-23	5.4e-19	2.9e-22
sub15	*r*	0.17	0.28	0.33	0.23	0.17	0.16	**0.12**
	p	1.5e-02	5.2e-05	1.3e-06	1.1e-03	1.8e-02	2.6e-02	**9.0e-02**
sub16	*r*	0.18	0.30	0.33	0.35	0.30	0.26	0.17
	p	1.5e-02	3.0e-05	3.6e-06	1.5e-06	2.7e-05	4.2e-04	1.8e-02

Table S9 The r and two-sided p_{FDR} values for the correlations between BOLD and SEEG FC evaluated using a window size of 4 s for SEEG analysis. The distance was regressed out from both FC matrices before evaluating the Spearman's rank correlation between them. False discovery rate (FDR) correction was applied to account for multiple comparisons across all 16 participants and all frequency bands. Non-significant (threshold: $p_{FDR} = 0.05$) correlations were labeled with bold font. BOLD: blood-oxygenation-level-dependent; SEEG: stereotactic EEG; FC: functional connectivity.

ID	r/p_{FDR}	**1-4Hz**	**4-8Hz**	**8-13Hz**	**13-30Hz**	**30-40Hz**	**40-70Hz**	**70-170Hz**
sub01	r	0.34	0.30	0.32	0.32	0.36	0.35	0.35
	p	1.2e-29	1.2e-22	2.0e-26	2.4e-25	1.9e-32	2.7e-30	2.7e-30
sub02	r	0.16	0.26	0.27	0.33	0.40	0.39	0.42
	p	1.1e-03	4.2e-08	2.4e-08	6.2e-12	2.5e-17	2.2e-16	2.0e-19
sub03	r	0.14	0.19	0.11	**0.03**	**0.06**	**0.02**	**0.03**
	p	4.8e-05	1.7e-08	1.2e-03	**3.4e-01**	**7.6e-02**	**4.9e-01**	**4.4e-01**
sub04	r	0.14	0.22	0.24	0.31	0.35	0.29	0.27
	p	4.2e-04	2.0e-08	1.4e-09	1.3e-14	7.9e-19	2.2e-13	1.1e-11
sub05	r	0.29	0.23	0.29	0.25	0.33	0.33	0.33
	p	4.6e-10	1.1e-06	4.6e-10	1.3e-07	2.0e-12	1.2e-12	7.2e-13
sub06	r	**-0.06**	**0.10**	0.22	0.31	0.35	0.20	**0.10**
	p	**3.2e-01**	**1.3e-01**	4.5e-04	8.7e-07	3.0e-08	1.7e-03	**1.3e-01**
sub07	r	0.07	0.12	0.15	0.14	0.12	0.13	0.10
	p	3.6e-04	2.8e-09	5.9e-14	1.4e-12	1.4e-08	3.1e-10	1.7e-06
sub08	r	0.22	0.26	0.12	**0.05**	0.14	0.14	0.20
	p	2.2e-07	9.0e-10	3.8e-03	**2.0e-01**	1.0e-03	1.2e-03	3.9e-06
sub09	r	0.16	0.26	0.32	0.40	0.40	0.32	0.29
	p	3.0e-03	7.8e-07	1.7e-09	2.3e-14	2.7e-14	1.4e-09	3.3e-08
sub10	r	0.34	0.32	0.37	0.35	0.34	0.34	0.34
	p	5.0e-09	1.6e-08	1.6e-10	8.0e-10	3.4e-09	3.8e-09	4.9e-09
sub11	r	0.14	0.15	0.14	0.21	0.19	0.19	0.18
	p	1.2e-09	7.9e-11	1.9e-09	6.5e-20	7.6e-16	8.0e-17	1.5e-15
sub12	r	0.28	0.33	0.19	0.29	0.37	0.28	0.21
	p	8.9e-22	2.3e-29	3.6e-10	7.2e-23	2.5e-37	3.9e-22	8.9e-13
sub13	r	**0.06**	0.10	0.15	0.26	0.24	0.28	0.27
	p	**9.5e-02**	1.1e-02	1.4e-04	5.6e-12	1.6e-10	2.2e-13	1.5e-12
sub14	r	0.19	0.27	0.41	0.52	0.59	0.49	0.54
	p	2.0e-03	6.7e-06	5.5e-12	6.5e-20	2.1e-26	3.4e-17	1.1e-21
sub15	r	**0.12**	0.28	0.31	0.20	**0.10**	0.20	**0.12**
	p	**1.0e-01**	4.0e-05	5.4e-06	3.7e-03	**1.4e-01**	4.6e-03	**8.7e-02**
sub16	r	0.22	0.28	0.30	0.33	0.20	0.27	0.17
	p	2.5e-03	1.5e-04	3.9e-05	4.1e-06	6.1e-03	2.1e-04	2.3e-02

Table S10 The *r* and two-sided p_{FDR} values for the correlation between BOLD and SEEG FC evaluated using a window size of 8 s for SEEG analysis. The distance was regressed out from both FC matrices before evaluating the Spearman's rank correlation between them. Non-significant (threshold: $p_{FDR} = 0.05$) correlations were labeled with bold font. False discovery rate (FDR) correction was applied to account for multiple comparisons across all 16 participants and all frequency bands. BOLD: blood-oxygenation-level-dependent; SEEG: stereotactic EEG; FC: functional connectivity.

ID	r/p_{FDR}	**1-4Hz**	**4-8Hz**	**8-13Hz**	**13-30Hz**	**30-40Hz**	**40-70Hz**	**70-170Hz**
sub01	*r*	0.41	0.36	0.30	0.35	0.35	0.33	0.35
	p	4.2e-43	4.5e-33	9.9e-23	7.3e-31	7.9e-31	1.6e-27	2.5e-31
sub02	*r*	0.14	0.26	0.27	0.32	0.39	0.37	0.40
	p	3.4e-03	4.2e-08	1.4e-08	3.1e-11	2.8e-16	1.3e-14	1.4e-17
sub03	*r*	0.14	0.20	0.13	**0.01**	**0.02**	**0.01**	**0.01**
	p	7.2e-05	4.2e-09	1.3e-04	**7.0e-01**	**5.2e-01**	**8.0e-01**	**7.2e-01**
sub04	*r*	0.16	0.25	0.19	0.37	0.37	0.29	0.27
	p	1.1e-04	2.6e-10	2.0e-06	6.0e-21	2.3e-21	1.8e-13	2.2e-11
sub05	*r*	0.29	0.27	0.24	0.29	0.33	0.35	0.31
	p	4.2e-10	5.0e-09	4.3e-07	3.0e-10	1.3e-12	2.3e-14	1.1e-11
sub06	*r*	**0.00**	**0.10**	0.13	0.29	0.26	0.20	**0.10**
	p	**9.7e-01**	**1.2e-01**	4.5e-02	2.8e-06	3.9e-05	1.4e-03	**1.2e-01**
sub07	*r*	0.10	0.11	0.11	0.10	0.10	0.13	0.10
	p	1.5e-06	1.3e-07	1.9e-07	1.1e-06	1.8e-06	5.4e-10	2.3e-06
sub08	*r*	0.21	0.26	0.17	0.09	0.25	0.18	0.20
	p	6.7e-07	1.4e-09	1.1e-04	3.3e-02	5.2e-09	2.6e-05	2.0e-06
sub09	*r*	0.23	0.28	0.30	0.46	0.45	0.35	0.29
	p	2.2e-05	2.1e-07	1.8e-08	6.9e-19	8.9e-18	5.3e-11	5.5e-08
sub10	*r*	0.23	0.32	0.32	0.32	0.27	0.26	0.20
	p	1.1e-04	3.1e-08	2.2e-08	3.9e-08	3.2e-06	7.5e-06	6.9e-04
sub11	*r*	0.17	0.19	0.16	0.24	0.17	0.19	0.18
	p	6.7e-13	7.3e-16	1.8e-12	1.2e-26	6.3e-14	3.9e-16	2.3e-15
sub12	*r*	0.27	0.27	0.21	0.33	0.33	0.27	0.24
	p	6.9e-21	1.7e-20	1.2e-12	2.3e-29	7.9e-31	2.2e-20	3.0e-16
sub13	*r*	0.16	0.11	0.14	0.24	0.29	0.30	0.29
	p	2.7e-05	3.1e-03	2.1e-04	2.1e-10	3.6e-14	1.3e-15	2.1e-14
sub14	*r*	0.18	0.30	0.39	0.50	0.61	0.52	0.54
	p	3.4e-03	4.8e-07	2.7e-11	6.7e-18	7.7e-29	3.7e-20	1.3e-21
sub15	*r*	0.23	0.26	0.28	0.26	0.17	0.16	**0.10**
	p	1.1e-03	1.8e-04	4.3e-05	1.8e-04	1.3e-02	1.9e-02	**1.6e-01**
sub16	*r*	0.20	0.27	0.33	0.32	0.30	0.27	**0.14**
	p	7.4e-03	2.1e-04	4.8e-06	7.5e-06	2.8e-05	2.2e-04	**5.1e-02**

Table S11 The r and two-sided p_{FDR} values for the correlations between BOLD FC and coherence-based SEEG FC. The distance was regressed out from both FC matrices before evaluating the Spearman's rank correlation between them. Non-significant (threshold: p_{FDR} = 0.05) correlations were labeled with bold font. False discovery rate (FDR) correction was applied to account for multiple comparisons across all 16 participants and all frequency bands. BOLD: blood-oxygenation-level-dependent; SEEG: stereotactic EEG; FC: functional connectivity.

ID	r/p_{FDR}	**1-4Hz**	**4-8Hz**	**8-13Hz**	**13-30Hz**	**30-40Hz**	**40-70Hz**	**70-170Hz**
sub01	*r*	0.19	0.34	0.30	0.32	0.26	0.33	0.31
	p	6.9e-10	1.2e-30	9.5e-23	2.3e-26	3.8e-17	6.1e-28	1.6e-24
sub02	*r*	0.19	0.15	0.16	0.18	0.24	0.22	0.29
	p	8.7e-05	2.0e-03	1.2e-03	2.2e-04	1.2e-06	8.7e-06	2.2e-09
sub03	*r*	0.14	**0.01**	0.10	0.14	0.20	0.18	0.14
	p	7.7e-05	**6.7e-01**	5.8e-03	3.4e-05	9.5e-09	1.8e-07	3.5e-05
sub04	*r*	0.32	0.32	0.39	0.35	0.32	0.36	0.43
	p	3.7e-16	1.2e-15	7.3e-24	1.6e-18	8.0e-16	1.5e-20	2.7e-29
sub05	*r*	0.37	0.20	0.25	0.23	0.29	0.18	0.17
	p	3.8e-16	1.8e-05	1.1e-07	9.9e-07	7.3e-10	1.6e-04	4.6e-04
sub06	*r*	0.25	0.31	0.17	0.23	0.24	0.31	0.25
	p	1.1e-04	6.4e-07	8.7e-03	3.0e-04	1.4e-04	6.4e-07	9.3e-05
sub07	*r*	0.15	0.20	0.21	0.25	0.31	0.24	0.22
	p	3.0e-14	1.4e-22	6.1e-25	2.9e-35	4.4e-54	1.0e-31	1.1e-26
sub08	*r*	-0.09	**0.08**	0.21	0.24	0.17	0.18	0.14
	p	3.4e-02	**5.5e-02**	6.0e-07	3.2e-08	7.2e-05	3.6e-05	9.0e-04
sub09	*r*	0.25	0.20	0.30	0.26	0.24	0.29	0.34
	p	3.0e-06	2.8e-04	1.4e-08	1.0e-06	1.4e-05	9.9e-08	2.1e-10
sub10	*r*	0.17	-0.12	**-0.04**	**0.04**	**0.09**	**0.11**	**0.08**
	p	4.7e-03	4.1e-02	**4.7e-01**	**4.5e-01**	**1.5e-01**	**6.7e-02**	**1.8e-01**
sub11	*r*	0.26	0.29	0.26	0.26	0.28	0.31	0.26
	p	1.0e-31	3.8e-38	1.6e-30	5.0e-31	8.7e-36	3.0e-44	1.5e-30
sub12	*r*	0.26	0.38	0.38	0.36	0.40	0.40	0.40
	p	2.1e-18	5.4e-41	5.7e-40	5.2e-36	1.4e-44	3.7e-45	3.2e-44
sub13	*r*	0.27	0.26	0.32	0.29	0.29	0.31	0.30
	p	5.4e-13	1.2e-11	4.7e-18	1.2e-14	8.0e-15	2.9e-16	1.1e-15
sub14	*r*	0.21	0.24	0.32	0.33	0.40	0.44	0.39
	p	6.5e-04	7.9e-05	7.3e-08	2.8e-08	7.4e-12	5.1e-14	2.5e-11
sub15	*r*	0.25	0.14	0.20	0.17	0.23	0.24	0.24
	p	4.4e-04	4.6e-02	5.2e-03	1.8e-02	8.4e-04	4.8e-04	5.1e-04
sub16	*r*	0.18	0.27	0.18	0.20	0.24	0.25	0.26
	p	1.3e-02	2.8e-04	1.2e-02	5.8e-03	8.5e-04	6.5e-04	3.2e-04

Table S12 The r and two-sided p_{FDR} values for the correlations between BOLD FC and structural connectivity. The distance was regressed out from both FC and structural connectivity before evaluating the Spearman's rank correlation between them. Non-significant (threshold: $p_{FDR} = 0.05$) correlations were labeled with bold font. False discovery rate (FDR) correction was applied to account for multiple comparisons across all 16 participants and all frequency bands. BOLD: blood-oxygenation-level-dependent; FC: functional connectivity.

ID	sub01	sub02	sub03	sub04	sub05	sub06	sub07	sub08
r	0.16	0.39	0.40	0.39	0.22	0.27	0.33	**0.12**
p_{FDR}	4.8e-04	8.7e-05	1.7e-09	1.3e-06	1.5e-03	6.7e-03	9.6e-24	**2.7e-01**
ID	sub09	sub10	sub11	sub12	sub13	sub14	sub15	sub16
r	0.44	0.62	0.17	0.49	0.27	0.33	0.25	**0.05**
p_{FDR}	4.3e-06	1.8e-07	1.7e-04	7.3e-08	1.5e-02	3.9e-03	4.3e-02	**5.6e-01**

Table S13 The *r* and two-sided p_{FDR} values for the correlations between SEEG FC and structural connectivity. The distance was regressed out from both FC and structural connectivity before evaluating the Spearman's rank correlation between them. Non-significant (threshold: $p_{FDR} = 0.05$) correlations were labeled with bold font. False discovery rate (FDR) correction was applied to account for multiple comparisons across all 16 participants and all frequency bands. SEEG: stereotactic EEG; FC: functional connectivity.

ID	r/p_{FDR}	**1-4Hz**	**4-8Hz**	**8-13Hz**	**13-30Hz**	**30-40Hz**	**40-70Hz**	**70-170Hz**
sub01	*r*	0.36	0.23	0.35	0.33	0.29	0.29	0.29
	p	5.6e-15	4.8e-07	5.6e-15	1.5e-13	2.0e-10	3.3e-10	3.7e-10
sub02	*r*	**0.18**	0.31	0.32	0.46	0.34	0.37	0.31
	p	**8.1e-02**	2.6e-03	1.9e-03	3.6e-06	6.9e-04	2.4e-04	2.2e-03
sub03	*r*	0.21	0.23	0.35	0.35	0.39	0.35	0.32
	p	2.2e-03	6.8e-04	1.7e-07	2.3e-07	7.0e-09	2.3e-07	1.6e-06
sub04	*r*	0.27	**0.13**	0.28	0.23	0.21	0.19	0.20
	p	8.8e-04	**1.0e-01**	5.7e-04	5.0e-03	9.3e-03	1.8e-02	1.2e-02
sub05	*r*	**0.10**	0.18	0.30	0.31	0.26	0.29	0.22
	p	**1.8e-01**	9.7e-03	1.3e-05	1.2e-05	2.8e-04	3.4e-05	1.5e-03
sub06	*r*	0.29	0.29	0.46	0.53	0.59	0.50	0.27
	p	4.1e-03	3.9e-03	2.6e-06	2.4e-08	3.3e-10	2.8e-07	7.4e-03
sub07	*r*	**-0.04**	**-0.01**	0.09	0.16	0.10	0.10	0.12
	p	**3.1e-01**	**6.9e-01**	1.1e-02	2.0e-06	2.5e-03	2.6e-03	4.7e-04
sub08	*r*	**0.14**	**0.05**	**-0.05**	**0.04**	**-0.01**	**-0.04**	**-0.05**
	p	**2.2e-01**	**6.7e-01**	**6.6e-01**	**7.3e-01**	**9.1e-01**	**7.0e-01**	**6.6e-01**
sub09	*r*	0.21	0.23	0.33	0.37	0.34	0.32	0.31
	p	3.5e-02	2.0e-02	6.4e-04	1.4e-04	4.7e-04	9.1e-04	1.6e-03
sub10	*r*	0.44	**0.20**	**0.16**	0.33	0.47	0.49	0.47
	p	5.3e-04	**1.3e-01**	**2.2e-01**	9.7e-03	2.5e-04	1.1e-04	2.3e-04
sub11	*r*	0.24	0.25	0.20	0.28	0.26	0.26	0.28
	p	2.3e-07	4.0e-08	1.2e-05	8.3e-10	1.8e-08	1.8e-08	9.6e-10
sub12	*r*	**0.13**	0.28	0.32	0.35	0.42	0.42	0.38
	p	**1.8e-01**	2.7e-03	7.5e-04	1.8e-04	4.7e-06	6.6e-06	4.1e-05
sub13	*r*	0.28	0.44	0.45	0.37	0.28	0.33	0.31
	p	1.0e-02	4.8e-05	2.6e-05	6.4e-04	9.7e-03	2.6e-03	4.0e-03
sub14	*r*	**0.18**	**0.13**	0.27	0.40	0.37	0.35	0.33
	p	**1.3e-01**	**2.7e-01**	2.0e-02	5.7e-04	1.1e-03	2.6e-03	4.1e-03
sub15	*r*	0.75	0.63	0.70	0.81	0.74	0.80	0.79
	p	3.6e-12	5.7e-08	3.3e-10	5.6e-15	4.9e-12	5.6e-15	1.5e-14
sub16	*r*	0.44	0.43	0.25	0.28	0.31	0.27	0.33
	p	5.3e-07	1.6e-06	5.8e-03	2.2e-03	5.7e-04	2.8e-03	3.2e-04

Supplementary References

1 Mori, S. *et al.* Stereotaxic white matter atlas based on diffusion tensor imaging in an ICBM template. *Neuroimage* **40**, 570-582, doi:10.1016/j.neuroimage.2007.12.035 (2008).

2 Oishi, K. *et al.* Human brain white matter atlas: identification and assignment of common anatomical structures in superficial white matter. *Neuroimage* **43**, 447-457, doi:10.1016/j.neuroimage.2008.07.009 (2008).

3 Betzel, R. F. *et al.* Structural, geometric and genetic factors predict interregional brain connectivity patterns probed by electrocorticography. *Nat Biomed Eng* **3**, 902-916, doi:10.1038/s41551-019-0404-5 (2019).

4 Mišić, B. *et al.* The Functional Connectivity Landscape of the Human Brain. *PLoS One* **9**, e111007, doi:10.1371/journal.pone.0111007 (2014).

5 Betzel, R. F. & Bassett, D. S. Specificity and robustness of long-distance connections in weighted, interareal connectomes. *Proc Natl Acad Sci U S A* **115**, E4880-E4889, doi:10.1073/pnas.1720186115 (2018).

6 Satterthwaite, T. D. *et al.* An improved framework for confound regression and filtering for control of motion artifact in the preprocessing of resting-state functional connectivity data. *Neuroimage* **64**, 240-256, doi:10.1016/j.neuroimage.2012.08.052 (2013).

7 Tadel, F., Baillet, S., Mosher, J. C., Pantazis, D. & Leahy, R. M. Brainstorm: a user-friendly application for MEG/EEG analysis. *Comput Intell Neurosci* **2011**, 879716, doi:10.1155/2011/879716 (2011).

8 Xia, M., Wang, J. & He, Y. BrainNet Viewer: a network visualization tool for human brain connectomics. *PLoS One* **8**, e68910, doi:10.1371/journal.pone.0068910 (2013).
