# Supplementary material for: Intracranial electrophysiological and structural basis of BOLD functional connectivity in human brain white matter
Source: Nat Commun. 2023 Jun 9;14:3414. doi: 10.1038/s41467-023-39067-3 (PMC10256794; doi:10.1038/s41467-023-39067-3)
Supplement: Supplementary file 3 — Reporting Summary [file 41467_2023_39067_MOESM3_ESM.pdf]

nature portfolio

Corresponding author(s): Zaixu Cui

Last updated by author(s): May 17, 2023

Reporting Summary

Nature Portfolio wishes to improve the reproducibility of the work that we publish. This form provides structure for consistency and transparency in reporting. For further information on Nature Portfolio policies, see our Editorial Policies and the Editorial Policy Checklist.

Statistics

For all statistical analyses, confirm that the following items are present in the figure legend, table legend, main text, or Methods section.

n/a	Confirmed	
☐	☒	The exact sample size (*n*) for each experimental group/condition, given as a discrete number and unit of measurement
☐	☒	A statement on whether measurements were taken from distinct samples or whether the same sample was measured repeatedly
☐	☒	The statistical test(s) used AND whether they are one- or two-sided<br>*Only common tests should be described solely by name; describe more complex techniques in the Methods section.*
☐	☒	A description of all covariates tested
☐	☒	A description of any assumptions or corrections, such as tests of normality and adjustment for multiple comparisons
☐	☒	A full description of the statistical parameters including central tendency (e.g. means) or other basic estimates (e.g. regression coefficient) AND variation (e.g. standard deviation) or associated estimates of uncertainty (e.g. confidence intervals)
☐	☒	For null hypothesis testing, the test statistic (e.g. *F*, *t*, *r*) with confidence intervals, effect sizes, degrees of freedom and *P* value noted<br>*Give P values as exact values whenever suitable.*
☒	☐	For Bayesian analysis, information on the choice of priors and Markov chain Monte Carlo settings
☒	☐	For hierarchical and complex designs, identification of the appropriate level for tests and full reporting of outcomes
☐	☒	Estimates of effect sizes (e.g. Cohen's *d*, Pearson's *r*), indicating how they were calculated

Our web collection on statistics for biologists contains articles on many of the points above.

Software and code

Policy information about availability of computer code

Data collection	The data were collected from patients at Xuanwu hospital with drug-resistant epilepsy who needed stereotaxic EEG (SEEG) monitoring to identify the precise origin of seizures. The electrodes (ALCIS, Besancon, France) were placed using a ROSA robot system (ROSA, Medtech, Montpellier, France) based on preoperative enhanced MRI images to avoid vascular injury. The contacts of the SEEG electrodes were cylinders of platinum-iridium alloy, 2 mm in length and 0.8 mm in diameter. The center-to-center space between the contacts was 3.5 mm, and each electrode comprised 5-15 contacts.<br>All MRI data, including structural MRI, BOLD fMRI, and DSI, were acquired using a GE Premier 3-T MRI scanner (General Electric Healthcare, Waukesha, WI, USA) with a 64-channel head coil at Xuanwu Hospital.
Data analysis	The MRI data were preprocessed using fMRIPrep 20.2.1, xcpd 0.0.9, QSIPrep 0.13.0RC2 and DSI studio. The SEEG data were preprocessed using Brainstorm, FreeSurfer 6.0.1, SPM12 and Fieldtrip. Network construction and analysis were carried out using custom MATLAB(R2019a) code. Analysis code is available here: https://github.com/CuiLabCIBR/IEEGwmFC, with a detailed explanation in the following link: https://github.com/CuiLabCIBR/IEEGwmFC/wiki.

For manuscripts utilizing custom algorithms or software that are central to the research but not yet described in published literature, software must be made available to editors and reviewers. We strongly encourage code deposition in a community repository (e.g. GitHub). See the Nature Portfolio guidelines for submitting code & software for further information.

Data

Policy information about availability of data

All manuscripts must include a data availability statement. This statement should provide the following information, where applicable:

- Accession codes, unique identifiers, or web links for publicly available datasets
- A description of any restrictions on data availability
- For clinical datasets or third party data, please ensure that the statement adheres to our policy

All the data required to reproduce our findings have been made publicly available (https://github.com/CuiLabCIBR/IEEGwmFC/tree/main/data), including BOLD and SEEG functional connectivity, structural connectivity, and the distance matrix for all the participants. The relevant data for visualizing the figures are provided as Source Data files. Raw data is available from the corresponding authors upon request.

Human research participants

Policy information about studies involving human research participants and Sex and Gender in Research.

Reporting on sex and gender	We reported that there were 9 males and 7 females in this study based on self-reported sex. We did not specially consider sex in the study design and did not analyze it. We also did not include it as a covariate as we performed individual-level analyses. Individual's sex information was provided in Table S1 and consent has been obtained to share this information.
Population characteristics	The 16 participants have a history of drug-resistance epilepsy. The participants ranged in age from 19 to 37 years with a mean age of 28.2 years and a standard deviation (SD) of 4.9 years; this sample included 9 males and 7 females.
Recruitment	From a database of 84 participants, we selected 16 participants with complete data in clinical SEEG recordings as well as preoperative structural, diffusion, functional MRIs, and post-surgery X-ray CT. There are no self-selection biases here.
Ethics oversight	All participants provided informed consent, and all study procedures were approved by the Institutional Review Boards of Xuanwu Hospital.

Note that full information on the approval of the study protocol must also be provided in the manuscript.

Field-specific reporting

Please select the one below that is the best fit for your research. If you are not sure, read the appropriate sections before making your selection.

☒ Life sciences ☐ Behavioural & social sciences ☐ Ecological, evolutionary & environmental sciences

For a reference copy of the document with all sections, see nature.com/documents/nr-reporting-summary-flat.pdf

Life sciences study design

All studies must disclose on these points even when the disclosure is negative.

Sample size	The population consisted of 16 individuals with medication resistant epilepsy. No sample size calculation was performed. This sample size is enough for our work as our study performed the analyses for each individual separately rather than at group level.
Data exclusions	From a database of 84 participants, we selected 16 participants who had complete data in clinical SEEG recordings as well as preoperative structural, diffusion, functional MRIs, and post-surgery X-ray computed tomography (CT).
Replication	Our analysis was performed for each individual separately rather than on a group level. We tested the correlation between BOLD and SEEG white matter functional connectivity as well as the correlation between white matter structural connectivity and functional connectivity for each individual. We found these results to be significant for each of the 16 individuals at most frequency bands. Therefore, our results were successfully replicated in each individual.
Randomization	There was no group allocation in this study.
Blinding	There was no group allocation in this study.

Reporting for specific materials, systems and methods

We require information from authors about some types of materials, experimental systems and methods used in many studies. Here, indicate whether each material, system or method listed is relevant to your study. If you are not sure if a list item applies to your research, read the appropriate section before selecting a response.

Materials & experimental systems

n/a	Involved in the study
☒	☐ Antibodies
☒	☐ Eukaryotic cell lines
☒	☐ Palaeontology and archaeology
☒	☐ Animals and other organisms
☒	☐ Clinical data
☒	☐ Dual use research of concern

Methods

n/a	Involved in the study
☒	☐ ChIP-seq
☒	☐ Flow cytometry
☐	☒ MRI-based neuroimaging

Magnetic resonance imaging

Experimental design

Design type: We analyzed two different MRI datasets: resting state functional MRI and diffusion MRI data.

Design specifications: Functional MRI data was collected at rest.

Behavioral performance measures: This study did not included any behavior measures.

Acquisition

Imaging type(s): fMRI, sMRI , dMRI

Field strength: 3.0 T

Sequence & imaging parameters: functional MRI: data were acquired with a gradient-echo echo planar imaging (EPI) sequence and parameters as follows: TR, 2000 ms; TE, 30 ms; FOV, 224 × 224 mm2; matrix, 64 × 64; slice thickness, 3.5 mm; the number of time points, 240; total scan time, 8 min.
Structural MRI: a magnetization-prepared, rapid acquisition gradient-echo (MPRAGE) T1-weighted image was acquired, with the following parameters: TR, 2477 ms; TE, 2.69 ms; FOV, 256×256 mm2; matrix, 256×256; 166 sagittal slices; slice thickness, 1mm with no gap; scanning duration, 6.8 min.
DSI: data acquisition was performed with 257 diffusion-weighted directions. The b-values ranged from 0 to 7000 s/mm2. The hyperband acceleration factor was 2 and other parameters were as follows: TR, 5548 ms; TE, 84.1 ms; voxel size, 2 mm×2 mm×2 mm. The total acquisition duration for the DSI sequence was 24 min.

Area of acquisition: Whole-Brain

Diffusion MRI: ☒ Used ☐ Not used

Parameters: Data acquisition was performed with 257 diffusion-weighted directions. The b-values (23 different b-values) ranged from 0 to 7000 s/mm2.

Preprocessing

Preprocessing software: fMRIPrep 20.2.1, xcpd 0.0.9, QSIPrep 0.13.0RC2 and fieldtrip

Normalization: Volume-based spatial normalization to MNI152NLin6Asym spaces was performed through nonlinear registration with ANTs 2.3.3.

Normalization template: the functional and structural MRI data were normalized into the MNI standard space.

Noise and artifact removal: The 24 motion parameters, including six frame-wise estimates of motion, the derivatives of each of these six parameters, and quadratic terms of each of the six parameters and their derivatives; global time series; CSF time series.

Volume censoring: Remove the first five volume of the fMRI data of every participant.

Statistical modeling & inference

Model type and settings: At the first level of analysis, we estimated the white matter functional connectivity matrices by evaluated the Pearson correlation between regional time series using both BOLD fMRI and intracranial SEEG data. We also generated the structural connectivity as the white matter streamline counts between two regions. At the second level of analysis, we used Spearman's rank correlation to evaluate the similarity between each two connectivity matrices.

Effect(s) tested: Pearson correlation was used to measure the strength of functional connectivity.

Specify type of analysis: ☐ Whole brain ☒ ROI-based ☐ Both

Anatomical location(s) | Brainstorm pipeline was used for anatomical localization of electrode contacts. According to ASEG atlas, we identified the contacts that localized in gray matter or white matter.

Statistic type for inference (See Eklund et al. 2016) | Spearman's rank correlation was used to evaluate the similarity between two connectivity matrices.

Correction | False discovery rate (FDR) correction was used to account for multiple comparisons.

Models & analysis

n/a	Involved in the study
☐	☒ Functional and/or effective connectivity
☒	☐ Graph analysis
☒	☐ Multivariate modeling or predictive analysis

Functional and/or effective connectivity | Functional connectivity was measured as the Pearson correlation between regional time series. Structural connectivity was defined as the streamline count between each pair of regions.
